# Foster’s Chemistry

**Published:** 1856-01

**Authors:** 


*Foster's Chemistry.*—This is an excellent little work for common schools, which we would be glad to see generally adopted.

